# Uncovering potential host proteins and pathways that may interact with eukaryotic short linear motifs in viral proteins of MERS, SARS and SARS2 coronaviruses that infect humans

**DOI:** 10.1371/journal.pone.0246150

**Published:** 2021-02-03

**Authors:** Chu-Wen Yang, Zhi-Ling Shi

**Affiliations:** 1 Department of Microbiology, Center for Applied Artificial Intelligence Research, Soochow University, Taipei, Taiwan; 2 Ocean School of Fuzhou University, Fuzhou University, Fuzhou, China; Loyola University Health System, UNITED STATES

## Abstract

A coronavirus pandemic caused by a novel coronavirus (SARS-CoV-2) has spread rapidly worldwide since December 2019. Improved understanding and new strategies to cope with novel coronaviruses are urgently needed. Viruses (especially RNA viruses) encode a limited number and size (length of polypeptide chain) of viral proteins and must interact with the host cell components to control (hijack) the host cell machinery. To achieve this goal, the extensive mimicry of SLiMs in host proteins provides an effective strategy. However, little is known regarding SLiMs in coronavirus proteins and their potential targets in host cells. The objective of this study is to uncover SLiMs in coronavirus proteins that are present within host cells. These SLiMs have a high possibility of interacting with host intracellular proteins and hijacking the host cell machinery for virus replication and dissemination. In total, 1,479 SLiM hits were identified in the 16 proteins of 590 coronaviruses infecting humans. Overall, 106 host proteins were identified that may interact with SLiMs in 16 coronavirus proteins. These SLiM-interacting proteins are composed of many intracellular key regulators, such as receptors, transcription factors and kinases, and may have important contributions to virus replication, immune evasion and viral pathogenesis. A total of 209 pathways containing proteins that may interact with SLiMs in coronavirus proteins were identified. This study uncovers potential mechanisms by which coronaviruses hijack the host cell machinery. These results provide potential therapeutic targets for viral infections.

## Introduction

Six coronaviruses have been known to infect humans. They are human coronavirus 229E (229E-CoV), human coronavirus NL63 (NL63-CoV), human coronavirus OC43 (OC43-CoV), human coronavirus HKU1 (HKU1-CoV), severe acute respiratory syndrome coronavirus (SARS-CoV) and Middle East respiratory syndrome coronavirus (MERS-CoV). HCoV-229E and HKU-NL63 are members of the Alphacoronavirus genus and are responsible for the common cold. HKU-OC43 and HCoV-HKU1 are Betacoronaviruses that cause self-limiting upper respiratory tract infections. SARS-CoV and MERS-CoV cause severe lower respiratory tract infections [1, 2]. A coronavirus pandemic caused by severe acute respiratory syndrome coronavirus 2 (SARS-CoV-2) has been spreading rapidly worldwide since December 2019. Over 194,000 confirmed cases have been identified up to March 2020 [3]. Increased understanding and new strategies to cope with novel coronaviruses are urgently needed.

Short linear motifs (SLiMs) are short protein sequences (3–10 residues) that can mediate protein-protein interactions. Three characteristics differentiate SLiMs from the global domains in proteins [4, 5]. The first feature of SLiMs is a functional interaction interface encoded in a short and poorly conserved sequence. The short length of the motifs makes them easy to arise/disappear via mutations (i.e., to appear de novo in unrelated protein sequences). The second characteristic of SLiMs is to increase the richness of functional motifs within a given length of protein sequence. The third characteristic of SLiMs is that the interactions mediated by SLiMs tend to be transient and have low binding affinities because a small number of residues are involved. They are ideal for mediating fast protein-protein interactions, such as interactions between phosphorylation sites on their binding partners in signal transduction pathways. These characteristics provide a flexible molecular basis for rapidly evolved proteins of RNA viruses to produce high versatility.

Viruses (especially RNA viruses) encode a limited number and size (length of polypeptide chain) of viral proteins that must establish complex networks of interactions with the host cell components to control (hijack) the host cell machinery. To achieve this goal, the extensive mimicry of host protein SLiMs provides an effective strategy [6–9]. Studies such as the identification of SLiMs in influenza A virus ribonucleoproteins [10] and the identification of protein-protein interactions between HIV-1 and human proteins mediated by SLiMs [11] have been reported. However, little is known regarding SLiMs in coronavirus proteins and their potential targets in host cells. The objective of this study is to uncover SLiMs in coronavirus proteins that are present in intracellular parts of human cells. These SLiMs have a high possibility of interacting with human intracellular proteins and hijacking the human cell machinery for virus replication and dissemination.

## Materials and methods

### Data sets

Sequences of fifteen nonstructural proteins (nsp1-10 and nsp12-16 from the open reading frame 1ab, orf1ab, of coronaviruses) and capsid proteins from 590 full-length genomes of coronaviruses isolated from human hosts were used. These proteins were studied because they perform their functions intracellularly in host cells. Therefore, SLiMs identified in these proteins may have the potential to interact with intracellular proteins of host cells, consequently affecting intracellular pathways in host cells. Protein sequences including 25 229E-CoVs, 60 NL63-CoVs, 39 HKU1-CoVs, 139 OC43-CoVs, 42 SARS-CoVs, 123 MERS-CoVs, 34 SARS-CoV-2s and 128 unclassified coronaviruses (other) were retrieved from the Virus Pathogen Resource (ViPR, https://www.viprbrc.org/) [12]. The accession numbers of virus genomes encoding the viral proteins used in this study are listed in S1 Table.

In total, 289 consensus sequences (regular expressions) of SLiMs and information on associated interaction proteins and pathways were retrieved from the Eukaryotic Linear Motif Resource for Functional Sites in Proteins (http://elm.eu.org/) [13].

### Data analysis

**Fig 1:**
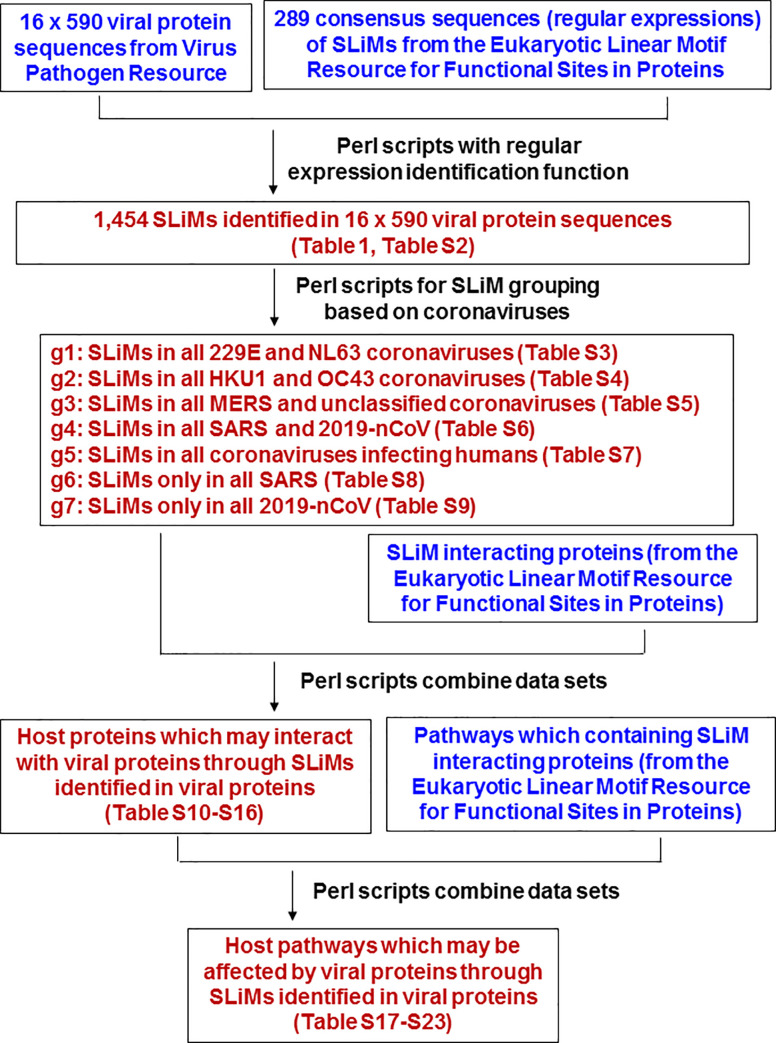
The data analysis procedures of this study. Blue words indicate input data. Brown words indicate the results of each step.

The data analysis procedures are shown in Fig 1. Sequence manipulation and SLiM identification (by regular expression matching) were performed using Perl scripts written by the authors. Phylogenetic analysis of orf1ab and capsid protein sequences was performed using ClustalX 2.1 using the neighbor-joining algorithm with 1,000 bootstrap replicates [14]. Heat maps were produced using the heatmap.2 function of the gplots package in R (https://www.r-project.org/). The website http://bioinformatics.psb.ugent.be/webtools/Venn/ was used to identify common and unique elements (intersection and union) among data sets.

## Results

### Identification of SLiMs in coronavirus proteins

**Fig 2:**
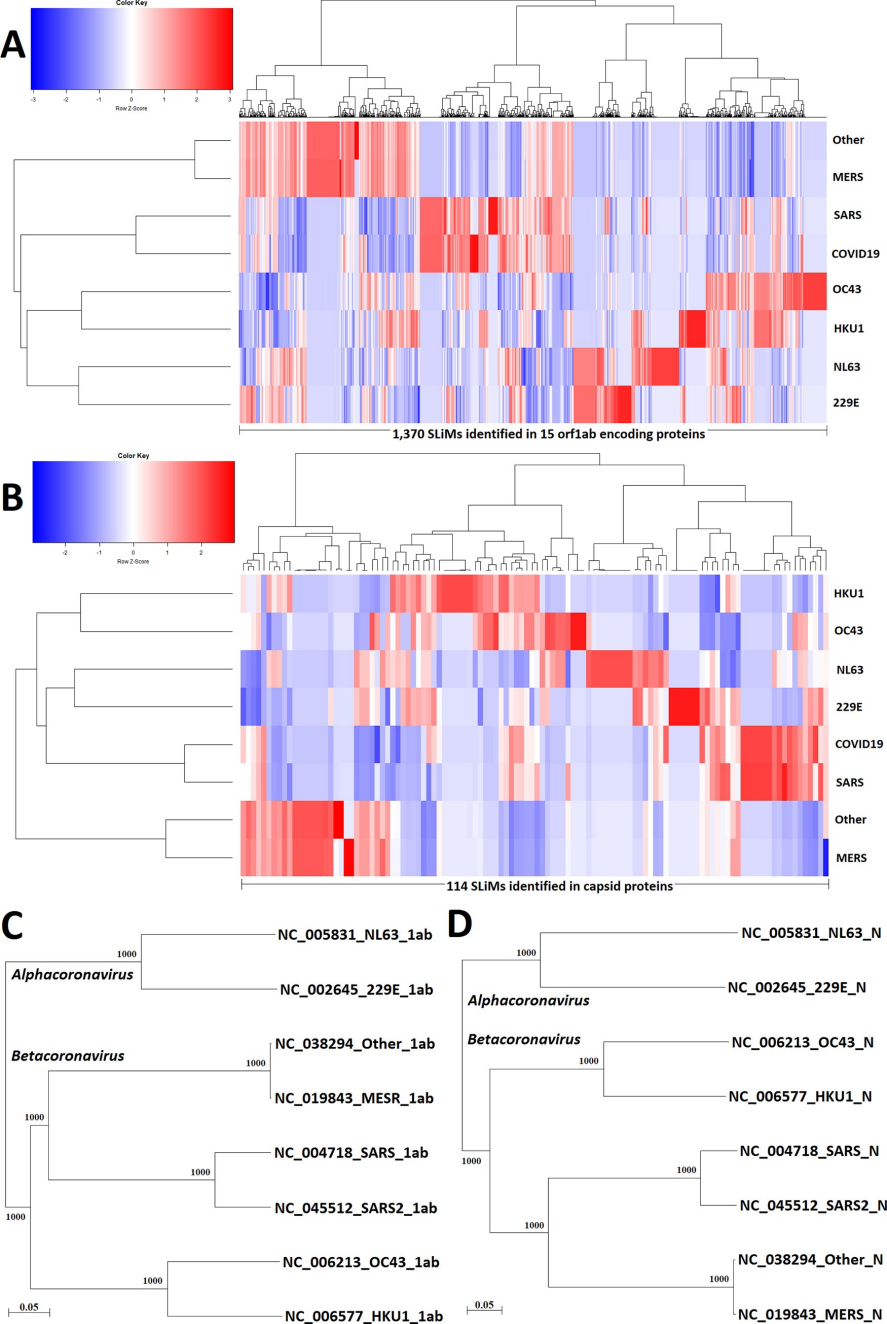
SLiM compositions and phylogenetic analysis of orf1ab-encoding proteins and capsid proteins of coronaviruses. (A) A total of 1,365 SLiMs were identified in 15 viral proteins encoded by orf1ab (nsp1-nsp10 and nsp12-nsp16). (B) A total of 114 SLiMs were identified in capsid proteins. Complete lists of SLiMs in (A) and (B) are shown in S2 Table. (C) Phylogenetic tree of representative orf1ab-encoding proteins. (D) Phylogenetic tree of representative capsid proteins. The number of tree branches that occurred in 1,000 bootstrap replicates are indicated at the branch point.

In total, 1,479 SLiM hits were identified in the 16 coronavirus proteins (Table 1). The complete results are listed in S2 Table. As shown in Fig 2, the topologies of SLiM composition trees (Fig 2A and 2B) and phylogenetic trees (protein sequence similarity) (Fig 2C and 2D) are different. These results indicate that SLiM compositions provide different information that could not be observed from the comparison of sequence similarity. According to the SLiM compositions of orf1ab-encoding proteins and capsid proteins, 590 coronaviruses were divided into four groups: (229E-CoV and NL63-CoV), (HKU1-CoV and OC43-CoV), (MERS-CoV and unclassified group in the ViPR database), and (SARS-CoV and SARS-CoV-2). As a consequence, seven groups (g1–g7) of SLiMs were separated from the 1,479 SLiM hits. Since one SLiM can be repeatedly identified in one and multiple viral proteins, nonredundant SLiMs of the seven groups are summarized in S3–S8 Tables.

**Table 1:** Number of SLiMs identified in coronavirus proteins.

Viral proteins	Viral protein function	g1	g2	g3	g4	g5	g6	g7	ALL
nsp1	suppress antiviral host response	2	1	1	4	4	4	2	98
nsp2	Unknown (promote viral RNA synthesis)	5	0	6	3	27	1	2	124
nsp3	putative Papain-like protease 2	3	2	1	3	55	2	1	148
nsp4	complex with nsp3 and 6: double-membrane vesicle formation	2	2	4	2	30	2	2	105
nsp5	3C-like protease	1	5	1	2	23	1	0	75
nsp6	complex with nsp3 and 4: double-membrane vesicles formation	3	1	4	2	19	1	0	91
nsp7	complex with nsp8: primase	5	0	7	4	3	0	0	49
nsp8	complex with nsp7: primase	8	2	2	3	12	0	0	68
nsp9	RNA/DNA binding activity	6	2	6	5	7	0	1	63
nsp10	complex with nsp14: replication fidelity	1	2	4	2	6	1	0	41
nsp12	RNA-dependent RNA polymerase	2	0	2	4	27	0	0	122
nsp13	helicase	2	2	5	5	34	0	0	105
nsp14	ExoN: 3’-5’ exoribonuclease	4	3	0	3	29	0	1	92
nsp15	XendoU: poly(U)-specific endoribonuclease	4	2	1	2	21	1	2	96
nsp16	2’-O-MT: 2’-O-ribose methyltransferase	0	2	0	3	16	0	0	88
N	Capsid protein	1	2	3	5	23	0	0	114
Total SLiM identified		49	28	47	52	336	13	11	1,479

ALL: number of all SLiMs identified in viral proteins in this study. g1: number of SLiMs (non-redundant) identified in viral proteins of all 229E and NL63 coronaviruses. g2: number of SLiMs (non-redundant) identified in viral proteins of all HKU1 and OC43 coronaviruses. g3: number of SLiMs (non-redundant) identified in viral proteins of all MERS and unclassified human coronaviruses. g4: number of SLiMs (non-redundant) identified in viral proteins of all SARS and SARS2. g5: number of SLiMs (non-redundant) identified in viral proteins of all coronaviruses that infecting humans. g6: number of SLiMs (non-redundant) identified only in viral proteins of all SARS. g7: number of SLiMs (non-redundant) identified only in viral proteins of all SARS2.

Group 1 (g1) SLiMs were composed of 42 SLiMs identified in proteins of all 229E-CoVs and NL63-CoVs but not identified in proteins of other coronaviruses (S3 Table). The group 2 (g2) SLiMs were composed of 26 SLiMs identified in proteins of all HKU1-CoVs and OC43-CoVs but not identified in proteins of other coronaviruses (S4 Table). The group 3 (g3) SLiMs were composed of 42 SLiMs identified in proteins of all MERS-CoVs and unclassified coronaviruses but not identified in proteins of other coronaviruses (S5 Table). The group 4 (g4) SLiMs were composed of 46 SLiMs identified in proteins of all SARS-CoVs and SARS-CoV-2s but not identified in proteins of other coronaviruses (S6 Table). The group 5 (g5) SLiMs were composed of 66 SLiMs identified in proteins of all 590 coronaviruses (S7 Table). The group 6 (g6) SLiMs were composed of 12 SLiMs identified in proteins of all SARS-CoVs but not identified in proteins of other coronaviruses (S8 Table). Group 7 (g7) SLiMs were composed of 11 SLiMs identified in proteins of all SARS-CoV-2s but not identified in proteins of other coronaviruses (S9 Table).

### Human proteins interacting with SLiMs identified in coronavirus proteins

**Fig 3:**
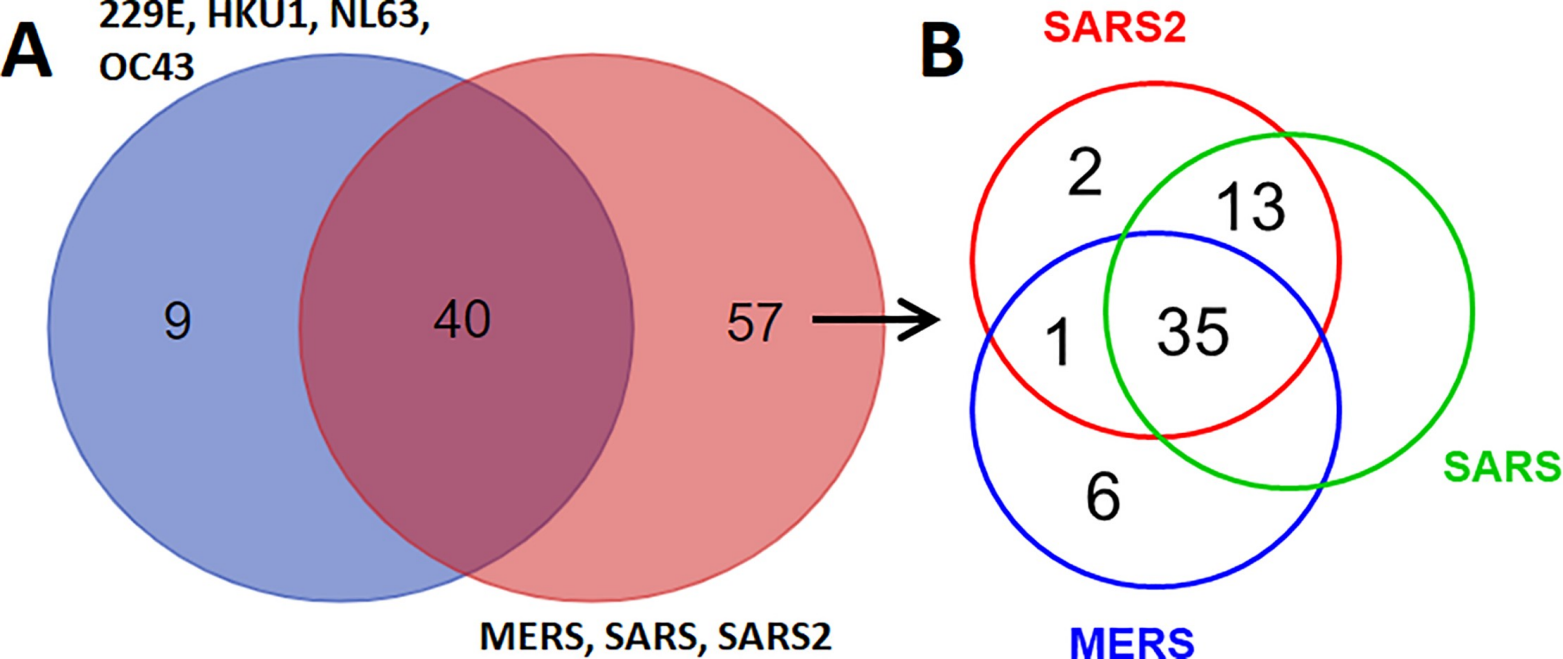
Venn diagram of number of human proteins interacting with coronavirus proteins mediated by SLiMs. (A) common and different SLiM interacting proteins between different human coronavirus groups. (B) common and different SLiM interacting proteins among SARS-CoV, SARS-CoV-2 and MERS-CoV.

To reveal the target host proteins that interact with SLiMs identified in coronavirus proteins, the information on SLiM interacting proteins was integrated into the seven groups of identified SLiMs and is shown in S10–S16 Tables. The common and different SLiM interacting proteins between different viral groups are summarized in Fig 3. Fifty-seven human proteins may interact with viral proteins of the SARS-CoV, SARS-CoV-2 and MERS-CoV groups mediated by SLiMs. Thirty-five of the fifty-seven human proteins interacting with viral proteins of SARS-CoV, SARS-CoV-2 and MERS-CoV are listed in Table 2. Another twenty-two of the fifty-seven human proteins interacting with viral proteins of SARS-CoV, SARS-CoV-2 or MERS-CoV are listed in Table 3. These results uncovered human intracellular proteins that may be specifically targeted by SARS-CoV, SARS-CoV-2 or MERS-CoV through SLiMs.

**Table 2:** Thirty-five human proteins interact with SLiMs identified in SARS-CoV, SARS-CoV-2 and MERS-CoV proteins.

IP Acc No	IP Name
P63104	14-3-3 protein zeta/delta
Q3YBA8	14-3-3 tau splice variant
P68133	Actin, alpha skeletal muscle
P48729	Casein kinase I isoform alpha
Q8N752	Casein kinase I isoform alpha-like
P48730	Casein kinase I isoform delta
P49674	Casein kinase I isoform epsilon
Q9HCP0	Casein kinase I isoform gamma-1
P78368	Casein kinase I isoform gamma-2
P68400	Casein kinase II subunit alpha
P06493	Cyclin-dependent kinase 1
P21127	Cyclin-dependent kinase 11B
P24941	Cyclin-dependent kinase 2
Q00526	Cyclin-dependent kinase 3
P11802	Cyclin-dependent kinase 4
Q00535	Cyclin-dependent-like kinase 5
P40692	DNA mismatch repair protein Mlh1
P03372	Estrogen receptor
Q92731	Estrogen receptor beta
P06730	Eukaryotic translation initiation factor 4E
Q9UKB1	F-box/WD repeat-containing protein 11
Q9BXW4	Microtubule-associated proteins 1A/1B light chain 3C
P27361	Mitogen-activated protein kinase 3
Q13526	Peptidyl-prolyl cis-trans isomerase NIMA-interacting 1
P12931	Proto-oncogene tyrosine-protein kinase Src
P06400	Retinoblastoma-associated protein
Q15172	Serine/threonine-protein phosphatase 2A 56 kDa regulatory subunit alpha isoform
Q15173	Serine/threonine-protein phosphatase 2A 56 kDa regulatory subunit beta isoform
Q14738	Serine/threonine-protein phosphatase 2A 56 kDa regulatory subunit delta isoform
Q16537	Serine/threonine-protein phosphatase 2A 56 kDa regulatory subunit epsilon isoform
Q13362	Serine/threonine-protein phosphatase 2A 56 kDa regulatory subunit gamma isoform
P40763	Signal transducer and activator of transcription 3
Q9Y5X1	Sorting nexin-9
Q9Y4K3	TNF receptor-associated factor 6
P61964	WD repeat-containing protein 5

**Table 3:** Twenty-two human proteins interact with SLiMs identified in SARS-CoV, SARS-CoV-2 or MERS-CoV proteins.

IP Acc No	IP Name
	**SARS-CoV, SARS-CoV-2**
P46108	Adapter molecule crk
P19784	Casein kinase II subunit alpha'
P46109	Crk-like protein
O95166	Gamma-aminobutyric acid receptor-associated protein
Q9H0R8	Gamma-aminobutyric acid receptor-associated protein-like 1
P60520	Gamma-aminobutyric acid receptor-associated protein-like 2
P49840	Glycogen synthase kinase-3 alpha
P49841	Glycogen synthase kinase-3 beta
Q9H492	Microtubule-associated proteins 1A/1B light chain 3A
Q9GZQ8	Microtubule-associated proteins 1A/1B light chain 3B
P27986	Phosphatidylinositol 3-kinase regulatory subunit alpha
P51955	Serine/threonine-protein kinase Nek2
P62136	Serine/threonine-protein phosphatase PP1-alpha catalytic subunit
	**MERS-CoV, SARS-CoV-2**
Q12834	Cell division cycle protein 20 homolog
	**SARS-CoV-2**
P03951	Coagulation factor XI
P03952	Plasma kallikrein
	**MERS-CoV**
Q07866	Kinesin light chain 1
Q8NHP6	Motile sperm domain-containing protein 2
O75340	Programmed cell death protein 6
Q12933	TNF receptor-associated factor 2
Q9P0L0	Vesicle-associated membrane protein-associated protein A
O95292	Vesicle-associated membrane protein-associated protein B/C

### Potential pathways may be targeted and affected by SLiMs in coronavirus proteins

**Fig 4:**
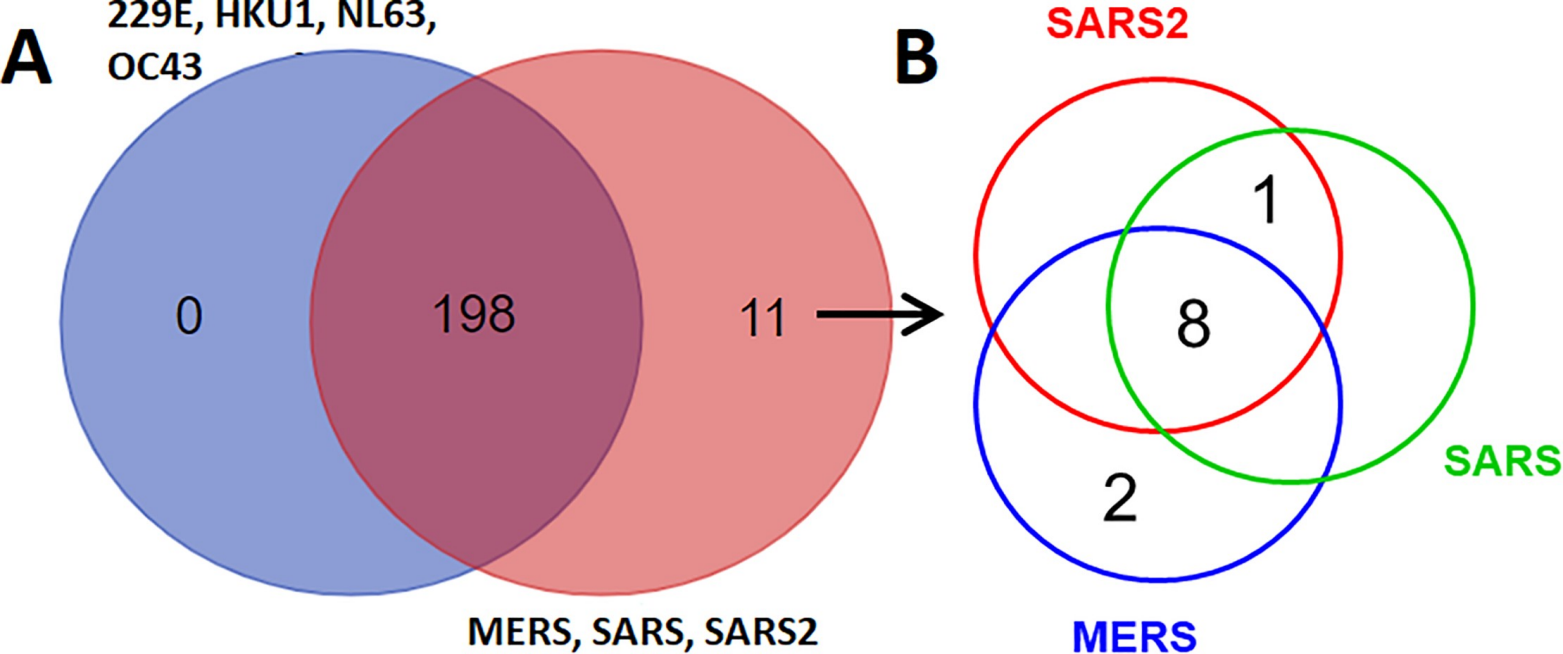
Venn diagram of the number of target human pathway coronavirus proteins mediated by SLiMs. (A) Common and different SLiM target pathways between different viral groups. (B) Common and different SLiM target pathways among SARS-CoV, SARS-CoV-2 and MERS-CoV.

To reveal the potential effects of SLiMs identified in viral proteins, information on SLiM-interacting protein-associated pathways was integrated into the seven groups of identified SLiMs and is shown in S17–S23 Tables. The common and different pathways targeted by different viral groups are summarized in Fig 4. Eleven pathways may be targeted by viral proteins of the SARS-CoV, SARS-CoV-2 and MERS-CoV groups mediated by SLiMs and are listed in Table 4. These pathways may be specifically targeted by SARS-CoV, SARS-CoV-2 or MERS-CoV through SLiMs.

**Table 4:** Pathways associated with viral SLiM interacting proteins.

Pathway id	Pathway
	**MERS-CoV, SARS-CoV, SARS-CoV-2**
hsa04340	Hedgehog signaling pathway
hsa03018	RNA degradation
hsa04973	Carbohydrate digestion and absorption
hsa04723	Retrograde endocannabinoid signaling
hsa04974	Protein digestion and absorption
hsa04970	Salivary secretion
hsa05310	Asthma
hsa04260	Cardiac muscle contraction
	**SARS-CoV, SARS-CoV-2**
hsa04140	Autophagy
	**MERS-CoV**
hsa00590	Arachidonic acid metabolism
hsa03010	Ribosome

Three pathways (hsa04340: Hedgehog signaling pathway, hsa04723: Retrograde endocannabinoid signaling and hsa04970: Salivary secretion) may be targeted by SLiMs that are present only in MERS-CoV, SARS-CoV and SARS-CoV-2 proteins (Table 2). Evidence is emerging indicating that the Hedgehog (Hh) signaling pathway is involved in postnatal processes such as tissue repair and adult immune responses. Therefore, Hh signaling has been reported as a target for some pathogens to control the local infected environment through the pathway [15, 16]. These studies indicate that select populations of immune cells are primed to respond to the Hh signal, often resulting in proliferation and upregulation of a subset of cytokines. Upregulated Hh signaling, as occurs in certain tissues during HBV, HCV, EBV, and HIV infections [17–20], may promote an environment for a modest replication shift to an environment that supports high-level replication. In contrast, limited pathway activation, such as influenza A virus infection, may suppress the immune response to evade it and/or protect the host from detrimental outcomes [21].

The retrograde endocannabinoid system (RECS) contains cannabinoid receptors CB1 and CB2. CB-2 receptors are most abundantly found in the immune system (spleen, tonsils, thymus glands and immune cells, including macrophages and leucocytes). The immediate effective actions of endocannabinoids on immune functions are due to location of immune cells and CB2 receptors throughout the body at localized sites [22]. Cannabinoids exhibit profoundly immunosuppressive effects on inflammatory and cellular antiviral responses. Many viral infection studies, both in vitro and in vivo, have demonstrated that cannabinoid treatment leads to disease progression, increased pathology, and sometimes host death [23]. Therefore, in many clinical settings, including acute infections caused by influenza A viruses and persistent infection of the liver caused by hepatitis C virus, cannabinoids lead to worsened disease outcome [24, 25]. Targeting retrograde endocannabinoid signaling by SLiMs in coronavirus proteins may be beneficial for coronavirus infections.

The salivary gland has been proposed as a target of SARS-CoV and SARS-CoV-2 infections because the angiotensin-converting enzyme 2 (ACE2) protein is expressed in the salivary gland duct epithelium [26, 27]. Several reports suggest the use of saliva for the diagnosis and monitoring of SARS-CoV and SARS-CoV-2 infections [28–30]. Targeting the salivary pathway by SLiMs in coronavirus proteins may be beneficial for coronavirus transmission.

### *In silico* validations

To validate the results of this study, a comprehensive in silico validation was performed using the data in the European Bioinformatics Institute (EMBL-EBI) Molecular Interaction Database (IntAct) (https://www.ebi.ac.uk/intact/) and BioGRID (v.3.5.187) Database (https://thebiogrid.org/). Three pairs of viral protein-host protein interactions identified in this study were found in IntAct: Replicase polyprotein 1ab (UniProt: P0C6X7) and Ubiquitin carboxyl-terminal hydrolase 7 (UniProt: Q93009) [31], capsid (UniProt: P59595) and SUMO-conjugating enzyme UBC9 (UniProt: P63279) [32] and capsid (UniProt: P33469) and Polyadenylate-binding protein 1 (UniProt: P11940) [33]. The results of this study were compared with the data in the BioGRID (v.3.5.187) Database (https://thebiogrid.org/), and another five pairs of viral protein-host protein interactions identified in this study were found in BioGRID: capsid (UniProt: P59595) and Cyclin-dependent kinase 2 (UniProt: P24941) [34], capsid (UniProt: P59595) and Glycogen synthase kinase-3 alpha (UniProt: P49840) [35], capsid (UniProt: P59595) and Caspase-3 (UniProt: P42574) [36], capsid (UniProt: P0DTC9) and Polyadenylate-binding protein 1 (UniProt: P11940) [37] and Replicase polyprotein 1ab (UniProt: P0C6X7) and Ubiquitin carboxyl-terminal hydrolase 7 (UniProt: Q93009) [31]. Two pairs of interactions, Replicase polyprotein 1ab (UniProt: P0C6X7) and Ubiquitin carboxyl-terminal hydrolase 7 (UniProt: Q93009) and capsid (UniProt: P0DTC9) and Polyadenylate-binding protein 1 (UniProt: P11940), are present in both databases. However, the two pairs of viral protein-host protein interactions were identified by different research reports [31, 33, 37, 38].

## Discussion

Viruses are obligate parasites completely dependent on host cells for replication and dissemination. Infection with viruses activates a series of host antiviral responses to inhibit viral replication and dissemination. Viruses have to evolve mechanisms to evade and subvert those host antiviral responses. Encoding SLiMs in viral proteins provides a solution to hijack, mimic and/or manipulate intracellular regulatory processes such as signal transduction, cell cycle, DNA damage repair and host immune responses [6–9]. It has been reported that SARS-CoV can induce G0/G1-phase arrest of infected host cells [38–41]. The SARS-CoV 3b nonstructural protein can induce cell cycle arrest at the G0/G1 phase [42]. Additionally, the SARS-CoV 7a nonstructural protein can inhibit cell growth and induce G0/G1-phase arrest. Expression of 7a was shown to decrease the levels of cyclin D3 and inhibit phosphorylation of pRb [38]. Moreover, many cell cycle-associated proteins, such as cyclin, cdk and E2Fs, were found to be targeted by proteins of RNA viruses [39–41]. Activation of the DNA damage response by RNA viruses (hepatitis C virus, influenza A virus, human immunodeficiency virus 1 and human T-cell lymphotropic virus 1) has been reported [43]. The Wnt pathway, a key pathway in cell signaling, has been reported to be dysregulated by DNA viruses such as Epstein-Barr virus, hepatitis B virus and human papillomavirus and RNA viruses, including hepatitis C virus and human immunodeficiency virus [44]. All of these proteins and pathways were identified in this study (Tables 2 and 3). The proteins listed in Table 2 provide more potential target proteins that may interact with SLiMs in coronavirus proteins. These SLiM-interacting proteins contain many key regulators, such as receptors (EGFR, PDGFRB, INSR, MET), transcription factors (TP53, JUN, MYC, RELA, RB1) and kinases (AKT1, MAP2K1, PTK2, RAF1), and may have important contributions to virus replication, immune evasion and viral pathogenesis.

The relationships between SLiMs, SLiM interacting proteins and SLiM interacting protein-associated pathways are multiple SLiMs to multiple interacting proteins to multiple pathways (a network) rather than a one-to-one connection (S17–S23 Tables). Although 26 SLiM-interacting proteins were identified only in SARS-CoV and/or SARS-CoV-2 proteins (Table 2, g4, g6 and g7) and 18 SLiM-interacting proteins were identified only in MERS-CoV proteins (Table 2, g3), there are very few pathways that are targeted specifically only by SLiMs identified in MERS-CoV, SARS-CoV and/or SARS-CoV-2. The results shown in S15 Table indicate that more potential interactions may exist between SLiMs in SARS-CoV proteins and their interacting proteins (such as JUN, NFATC1, CASP9, CREB3, PTEN, ROCK1, CD40, RB1, E2F1 and MAP2K4). In contrast, the results shown in S16 Table indicate that more potential interactions may exist between SLiMs in SARS-CoV-2 proteins and their interacting proteins (such as JUN, MYC, CCNE1, POLD3, HIF1A, CDC20, CDC25C, MAPKAPK2, PTTG1, SREBF1, etc.). As a consequence, different amounts and compositions of SLiMs in different types and strains of coronaviruses may lead to different strengths, different target sites and different effects on host intracellular pathways.

**Fig 5:**
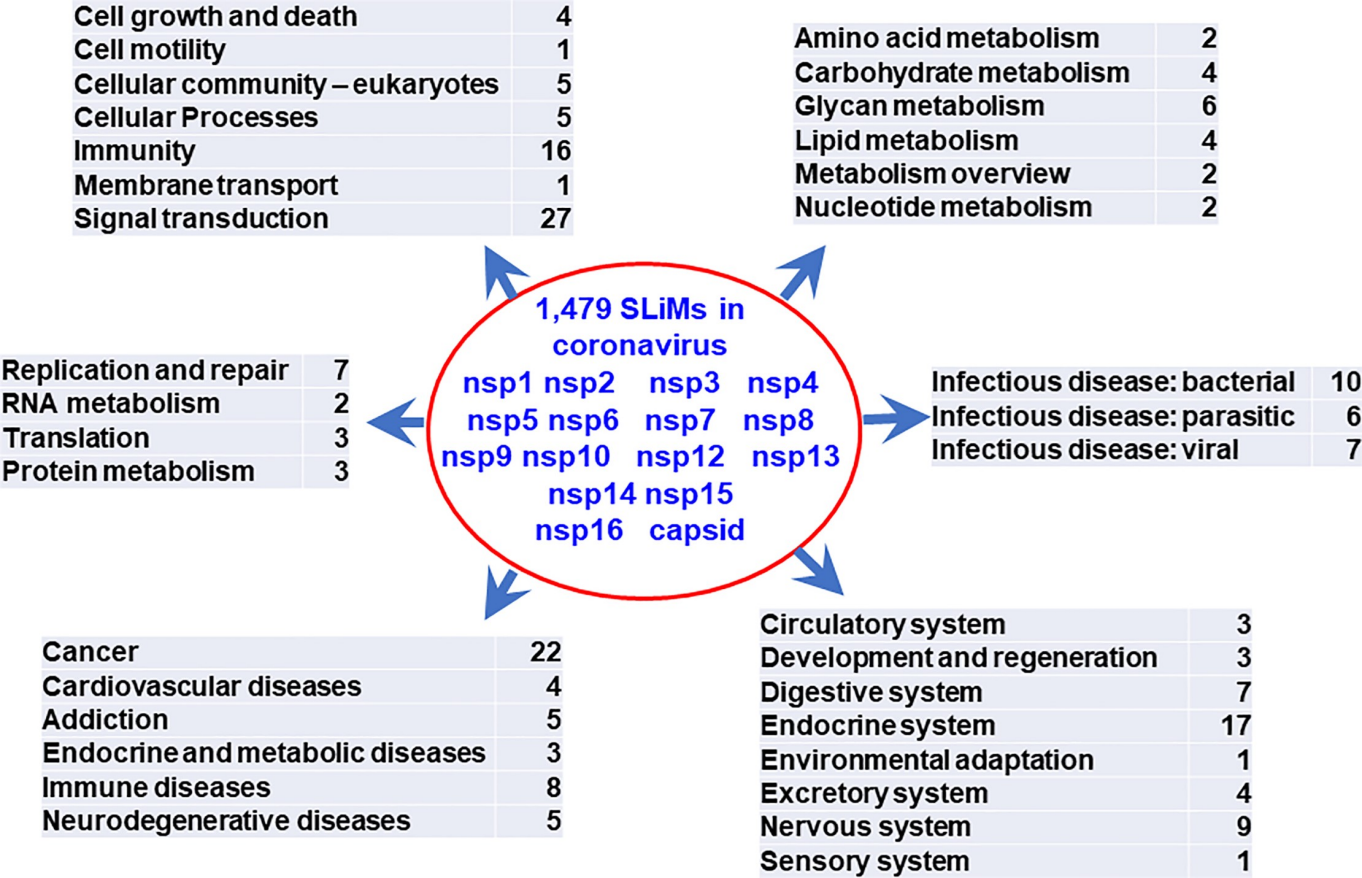
Categories (based on the KEGG pathway database) and numbers of pathways that may be affected by coronavirus proteins. Two hundred and nine pathways contain proteins that interact with 1,479 SLiMs identified in 16 coronavirus proteins (nsp1-nsp10, nsp12-nsp16 and capsid proteins).

Categories and numbers of pathways containing proteins interacting with SLiMs identified in 16 coronavirus proteins are summarized in Fig 5. Four cell growth and death pathways affected by coronavirus proteins have been reported [38–44]. Sixteen immunity pathways, 27 signal transduction pathways and 17 endocrine system-associated pathways suggest that multiple key regulator pathways in host cells may be targeted by viral proteins through SLiMs. Moreover, 26 infectious disease-associated pathways suggest that key regulatory pathways targeted by different pathogens may be common. Forty-seven noninfectious disease-associated pathways suggest that key regulators among these pathogenic pathways may be common. These potential key regulators (and may be potential therapeutic targets of viral infections) are present in the lists of SLiM interacting proteins in this study (Table 2 and S10–S16 Tables).

## Conclusion

This study revealed interacting proteins and associated pathways that may be targeted by coronavirus proteins by SLiMs. Different amounts and compositions of SLiMs in different types and strains of coronaviruses may lead to different target sites, different target strengths (variation in affinity of protein-protein interaction, number of target sites in a pathway), and as a consequence, different effects on human intracellular proteins and pathways. These results provide potential targets (virus-host protein interactions) to design antiviral strategies.

## Supporting information

S1 Table Number and accession numbers of coronavirus genome sequences used in this study. (DOCX)

S2 Table Number of SLiMs in different proteins of different coronaviruses. (XLSX)

S3 Table Group 1 SLiMs identified only in proteins of 229E-CoVs and NL63-CoVs. (XLSX)

S4 Table Group 2 SLiMs identified only in proteins of HKU1-CoVs and OC43-CoVs. (XLSX)

S5 Table Group 3 SLiMs identified only in proteins of MERS-CoVs and unclassified coronaviruses. (XLSX)

S6 Table Group 4 SLiMs identified only in proteins of SARS-CoVs and SARS-CoV-2s. (XLSX)

S7 Table Group 5 SLiMs identified only in proteins of all coronaviruses. (XLSX)

S8 Table Group 6 SLiMs identified only in proteins of SARS-CoVs. (XLSX)

S9 Table Group 7 SLiMs identified only in proteins of SARS-CoV-2s. (XLSX)

S10 Table Group 1 interacting proteins of SLiMs identified only in proteins of 229E-CoVs and NL63-CoVs. (XLSX)

S11 Table Group 2 interacting proteins of SLiMs identified only in proteins of HKU1-CoVs and OC43-CoVs. (XLSX)

S12 Table Group 3 interacting proteins of SLiMs identified only in proteins of MERS-CoVs and unclassified coronaviruses. (XLSX)

S13 Table Group 4 interacting proteins of SLiMs identified only in proteins of SARS-CoVs and SARS-CoV-2s. (XLSX)

S14 Table Group 5 interacting proteins of SLiMs identified only in proteins of all coronaviruses. (XLSX)

S15 Table Group 6 interacting proteins of SLiMs identified only in proteins of SARS-CoVs. (XLSX)

S16 Table Group 7 interacting proteins of SLiMs identified only in proteins of SARS-CoV-2s. (XLSX)

S17 Table Group 1 pathways associated with SLiMs identified only in proteins of 229E-CoVs and NL63-CoVs. (XLSX)

S18 Table Group 2 pathways associated with SLiMs identified only in proteins of HKU1-CoVs and OC43-CoVs. (XLSX)

S19 Table Group 3 pathways associated with SLiMs identified only in proteins of MERS-CoVs and unclassified coronaviruses. (XLSX)

S20 Table Group 4 pathways associated with SLiMs identified only in proteins of SARS-CoVs and SARS-CoV-2s. (XLSX)

S21 Table Group 5 pathways associated with SLiMs identified only in proteins of all coronaviruses. (XLSX)

S22 Table Group 6 pathways associated with SLiMs identified only in proteins of SARS-CoVs. (XLSX)

S23 Table Group 7 pathways associated with SLiMs identified only in proteins of SARS-CoV-2s. (XLSX)

## References

[1] Corman VM, Muth D, Niemeyer D, Drosten C. Hosts and Sources of Endemic Human Coronaviruses. Adv Virus Res. 2018 100:163–188. 10.1016/bs.aivir.2018.01.001 29551135 PMC7112090

[2] Abdoli A, Alirezaei M, Mehrbod P, Forouzanfar F. Autophagy: The multi-purpose bridge in viral infections and host cells. Rev Med Virol. 2018 28(4):e1973 10.1002/rmv.1973 29709097 PMC7169200

[3] Khan S, Siddique R, Shereen MA, Ali A, Liu J, Bai Q, et al The emergence of a novel coronavirus (SARS-CoV-2), their biology and therapeutic options. J Clin Microbiol. 2020 11 pii: JCM.00187-20. 10.1128/JCM.00187-20 32161092 PMC7180238

[4] Diella F, Haslam N, Chica C, Budd A, Michael S, Brown NP, et al Understanding eukaryotic linear motifs and their role in cell signaling and regulation. Front Biosci. 2008 13:6580–6603. 10.2741/3175 18508681

[5] Van Roey K, Uyar B, Weatheritt RJ, Dinkel H, Seiler M, Budd A, et al Short linear motifs: ubiquitous and functionally diverse protein interaction modules directing cell regulation. Chem Rev. 2014 114(13):6733–78. 10.1021/cr400585q 24926813

[6] Vidalain PO, Tangy F. Virus-host protein interactions in RNA viruses. Microbes Infect. 2010 12(14–15):1134–43. 10.1016/j.micinf.2010.09.001 20832499

[7] Davey NE, Travé G, Gibson TJ. How viruses hijack cell regulation. Trends Biochem Sci. 2011 36(3):159–69. 10.1016/j.tibs.2010.10.002 21146412

[8] Hagai T, Azia A, Babu MM, Andino R. Use of host-like peptide motifs in viral proteins is a prevalent strategy in host-virus interactions. Cell Rep. 2014 7(5):1729–1739. 10.1016/j.celrep.2014.04.052 24882001 PMC4089993

[9] Sobhy H. A Review of Functional Motifs Utilized by Viruses. Proteomes. 2016 4(1). pii: E3 10.3390/proteomes4010003 28248213 PMC5217368

[10] Yang CW. A comparative study of short linear motif compositions of the influenza A virus ribonucleoproteins. PLoS One. 2012 7(6):e38637 10.1371/journal.pone.0038637 22715401 PMC3371030

[11] Becerra A, Bucheli VA, Moreno PA. Prediction of virus-host protein-protein interactions mediated by short linear motifs. BMC Bioinformatics. 2017 18(1):163 10.1186/s12859-017-1570-7 28279163 PMC5345135

[12] Pickett BE, Greer DS, Zhang Y, Stewart L, Zhou L, Sun G, et al Virus pathogen database and analysis resource (ViPR): a comprehensive bioinformatics database and analysis resource for the coronavirus research community. Viruses. 2012 4(11):3209–26. 10.3390/v4113209 23202522 PMC3509690

[13] Kumar M, Gouw M, Michael S, Sámano-Sánchez H, Pancsa R, Glavina J, et al ELM-the eukaryotic linear motif resource in 2020. Nucleic Acids Res. 2020 48(D1):D296–D306. 10.1093/nar/gkz1030 31680160 PMC7145657

[14] Larkin MA, Blackshields G, Brown NP, Chenna R, McGettigan PA, McWilliam H, et al Clustal W and Clustal X version 2.0. Bioinformatics. 2007 23(21):2947–2948. 10.1093/bioinformatics/btm404 17846036

[15] Smelkinson MG. The Hedgehog Signaling Pathway Emerges as a Pathogenic Target. J Dev Biol. 2017 5(4):14 10.3390/jdb5040014 29214147 PMC5713906

[16] Crompton T, Outram SV, Hager-Theodorides AL. Sonic hedgehog signalling in T-cell development and activation. Nat Rev Immunol. 2007 7(9):726–735. 10.1038/nri2151 17690714

[17] Choi SS, Bradrick S, Qiang G, et al Up-regulation of Hedgehog pathway is associated with cellular permissiveness for hepatitis C virus replication. Hepatology. 2011 54(5):1580–1590. 10.1002/hep.24576 21793033 PMC3205266

[18] Pereira Tde A, Witek RP, Syn WK, et al Viral factors induce Hedgehog pathway activation in humans with viral hepatitis, cirrhosis, and hepatocellular carcinoma. Lab Invest. 2010 90(12):1690–1703. 10.1038/labinvest.2010.147 20697376 PMC2980808

[19] Lan X, Wen H, Cheng K, et al Hedgehog pathway plays a vital role in HIV-induced epithelial-mesenchymal transition of podocyte. Exp Cell Res. 2017 352(2):193–201. 10.1016/j.yexcr.2017.01.019 28159470 PMC6762036

[20] Deb Pal A, Banerjee S. Epstein-Barr virus latent membrane protein 2A mediated activation of Sonic Hedgehog pathway induces HLA class Ia downregulation in gastric cancer cells. Virology. 2015 484:22–32. 10.1016/j.virol.2015.05.007 26057149

[21] Smelkinson MG, Guichard A, Teijaro JR, et al Influenza NS1 directly modulates Hedgehog signaling during infection. PLoS Pathog. 2017 13(8):e1006588 10.1371/journal.ppat.1006588 28837667 PMC5587344

[22] Liu L, Wei Q, Alvarez X, et al Epithelial cells lining salivary gland ducts are early target cells of severe acute respiratory syndrome coronavirus infection in the upper respiratory tracts of rhesus macaques. J Virol. 2011 85(8):4025–4030. 10.1128/JVI.02292-10 21289121 PMC3126125

[23] Wang C, Wu H, Ding X, et al Does infection of 2019 novel coronavirus cause acute and/or chronic sialadenitis? Med Hypotheses. 2020 140:109789 10.1016/j.mehy.2020.109789 32361098 PMC7194735

[24] To KK, Tsang OT, Leung WS, et al Temporal profiles of viral load in posterior oropharyngeal saliva samples and serum antibody responses during infection by SARS-CoV-2: an observational cohort study. Lancet Infect Dis. 2020 20(5):565–574. 10.1016/S1473-3099(20)30196-1 32213337 PMC7158907

[25] Ceron JJ, Lamy E, Martinez-Subiela S, et al Use of Saliva for Diagnosis and Monitoring the SARS-CoV-2: A General Perspective. J Clin Med. 2020 9(5):1491 Published 2020 May 15. 10.3390/jcm9051491 32429101 PMC7290439

[26] Xu R, Cui B, Duan X, Zhang P, Zhou X, Yuan Q. Saliva: potential diagnostic value and transmission of 2019-nCoV. Int J Oral Sci. 2020 12(1):11 10.1038/s41368-020-0080-z 32300101 PMC7162686

[27] Oláh A, Szekanecz Z, Bíró T. Targeting Cannabinoid Signaling in the Immune System: "High"-ly Exciting Questions, Possibilities, and Challenges. Front Immunol. 2017 8:1487 Published 2017 Nov 10. 10.3389/fimmu.2017.01487 29176975 PMC5686045

[28] Boorman E, Zajkowska Z, Ahmed R, Pariante CM, Zunszain PA. Crosstalk between endocannabinoid and immune systems: a potential dysregulation in depression?. Psychopharmacology (Berl). 2016 233(9):1591–1604. 10.1007/s00213-015-4105-9 26483037 PMC4828487

[29] Tahamtan A, Tavakoli-Yaraki M, Rygiel TP, Mokhtari-Azad T, Salimi V. Effects of cannabinoids and their receptors on viral infections. J Med Virol. 2016 88(1):1–12. 10.1002/jmv.24292 26059175

[30] Reiss CS. Cannabinoids and Viral Infections. Pharmaceuticals (Basel). 2010 3(6):1873–1886. 10.3390/ph3061873 20634917 PMC2903762

[31] Cornillez-Ty CT, Liao L, Yates JR 3rd, Kuhn P, Buchmeier MJ. Severe acute respiratory syndrome coronavirus nonstructural protein 2 interacts with a host protein complex involved in mitochondrial biogenesis and intracellular signaling. J Virol. 2009 83(19):10314–10318. 10.1128/JVI.00842-09 19640993 PMC2748024

[32] Li Q, Xiao H, Tam JP, Liu DX. Sumoylation of the nucleocapsid protein of severe acute respiratory syndrome coronavirus by interaction with Ubc9. Adv Exp Med Biol. 2006 581:121–126. 10.1007/978-0-387-33012-9_21 17037517 PMC7123588

[33] Lai FW, Stephenson KB, Mahony J, Lichty BD. Human coronavirus OC43 nucleocapsid protein binds microRNA 9 and potentiates NF-κB activation. J Virol. 2014 88(1):54–65. 10.1128/JVI.02678-13 24109243 PMC3911702

[34] Surjit M, Liu B, Chow VT, Lal SK. The nucleocapsid protein of severe acute respiratory syndrome-coronavirus inhibits the activity of cyclin-cyclin-dependent kinase complex and blocks S phase progression in mammalian cells. J Biol Chem. 2006 281(16):10669–10681. 10.1074/jbc.M509233200 16431923 PMC7995956

[35] Wu CH, Yeh SH, Tsay YG, et al Glycogen synthase kinase-3 regulates the phosphorylation of severe acute respiratory syndrome coronavirus nucleocapsid protein and viral replication. J Biol Chem. 2009 284(8):5229–5239. 10.1074/jbc.M805747200 19106108 PMC8011290

[36] Ying W, Hao Y, Zhang Y, et al Proteomic analysis on structural proteins of Severe Acute Respiratory Syndrome coronavirus. Proteomics. 2004 4(2):492–504. 10.1002/pmic.200300676 14760722 PMC7168022

[37] Gordon DE, Jang GM, Bouhaddou M, et al A SARS-CoV-2 protein interaction map reveals targets for drug repurposing. Nature. 2020 10.1038/s41586-020-2286-9 32353859 PMC7431030

[38] Yuan X, Wu J, Shan Y, Yao Z, Dong B, Chen B, et al SARS coronavirus 7a protein blocks cell cycle progression at G0/G1 phase via the cyclin D3/pRb pathway. Virology. 2006 346(1):74–85. 10.1016/j.virol.2005.10.015 16303160 PMC7111786

[39] Chen CJ, Makino S. Murine coronavirus replication induces cell cycle arrest in G0/G1 phase. J Virol. 2004 78(11):5658–5669. 10.1128/JVI.78.11.5658-5669.2004 15140963 PMC415820

[40] Chen CJ, Sugiyama K, Kubo H, Huang C, Makino S. Murine coronavirus nonstructural protein p28 arrests cell cycle in G0/G1 phase. J Virol. 2004 78(19):10410–10419. 10.1128/JVI.78.19.10410-10419.2004 15367607 PMC516409

[41] Fan Y, Sanyal S, Bruzzone R. Breaking Bad: How Viruses Subvert the Cell Cycle. Front Cell Infect Microbiol. 2018 8:396 10.3389/fcimb.2018.00396 30510918 PMC6252338

[42] Yuan X, Shan Y, Zhao Z, Chen J, Cong Y. G0/G1 arrest and apoptosis induced by SARS-CoV 3b protein in transfected cells. Virol J. 2005 2:66 10.1186/1743-422X-2-66 16107218 PMC1190220

[43] Ryan EL, Hollingworth R, Grand RJ. Activation of the DNA Damage Response by RNA Viruses. Biomolecules. 2016 6(1):2 10.3390/biom6010002 26751489 PMC4808796

[44] van Zuylen WJ, Rawlinson WD, Ford CE. The Wnt pathway: a key network in cell signalling dysregulated by viruses. Rev Med Virol. 2016 26(5):340–55. 10.1002/rmv.1892 27273590

