# Supplementary material for: Uncovering potential host proteins and pathways that may interact with eukaryotic short linear motifs in viral proteins of MERS, SARS and SARS2 coronaviruses that infect humans
Source: PLoS One. 2021 Feb 3;16(2):e0246150. doi: 10.1371/journal.pone.0246150 (PMC7857568; doi:10.1371/journal.pone.0246150)
Supplement: S1 Table — (DOCX) [file pone.0246150.s001.docx]

Table S1. Number and accession numbers of coronavirus genome sequences used in this study.

| Viruses | Number of viruses | Accession Number of Genome sequences |
| --- | --- | --- |
| 229E | 25 | MF542265, KU291448, MN306046, KY369913, KY369910, KY369911, KY684760, KY967357, KY983587, KY621348, KY369908, KY369912, KY996417, KF514433, KF514430, JX503061, AF304460, NC_002645, JX503060, MN369046, KY369914, KF514432, KY674919, KY674914, KY369909 |
| NL63 | 60 | KU521535, KT381875, KX179500, JQ765566, KY554967, KY554968, KY674916, KY674915, KF530110, KY554970, JQ765573, JX504050, AY567487, NC_005831,KT266906, JQ765571, JQ765568, KF530105, JQ765572, JQ765575, KY554969, KY829118, KY554971, MK334045, DQ445911, KF530113, KF530104, MK334043, KF530109, KF530107, JQ765567, KF530108, KF530114, MK334044, KF530111, JQ765574, KF530106, JX524171, DQ445912, JQ765570, JQ765564, JQ765565, JQ765569, JQ765563, KF530112, MG772808, MK334047, MK334046, JX104161, MG428705, MG428701, MG428703, MG428706, MG428704, MG428699, MG428702, KY983586, MN306018, MN306040, AY518894 |
| HKU1 | 39 | KF686346, KF686343, KF430201, KF686342, KF686340, KY674941, KF686341, MH940245, KT779556, KT779555, KY674943, KY674942, MK167038, KY674921, NC_006577, KF686344, HM034837, DQ415901, AY597011, DQ415896, DQ415903, DQ415908, DQ415912, DQ415899, DQ415914, DQ415907, DQ415900, DQ415897, DQ415906, DQ415913, AY884001, DQ415910, DQ415909, DQ415905, DQ339101, DQ415898, DQ415904, DQ415911, DQ415902 |
| OC43 | 139 | MN306042, KF530089, KF530067, KF530082, KF530094, KF530079, KF530063, MN306041, KY684759, KY983583, KY967358, KY983585, KY369906, KY369905, MN306053, KY369907, KF530088, KF530096, KF530091, KF530095, KF530071, KF530084, KF530098, KF530099, KF530069, KY554972, KY554973, KY554974, KY554975, KF530092, KF530060, KF530085, KF530086, KF530077, KF530083, KF530087, KF530073, KF530066, KF530065, KF530061, KF530097, KF530074, KF530090, KF530075, KF530078, KF530064, KF530072, KF530080, KX344031, MN310476, KY983588, KY967359, KY967361, MN306036, KF530068, KF530070, KF530081, MN306043, MF374985, KY014282, MH121121, MF374983, MN026164, KY014281, MN310478, KP198611, KP198610, MF314143, KY967360, MG977452, JN129835, KU131570, KX538975, KX538977, KX538965, KX538968, KX538969, KX538971, KX538973, KX538974, MG977451, KJ958218, KJ958219, KX538964, KX538976, KX538978, KX538979, KX538966, KX538967, KX538970, KX538972, JN129834, MF374984, KY967356, FJ415324, KF530076, KF923895, KF923886, KF923889, KF923887, KF923888, KF923896, KF923906, KF923905, KF923898, KF923899, KF923900, KF923918, KF923924, KF923925, KF923902, KF923903, KF923904, KF923893, KF923897, KF923890, KF923891, KF923892, KF923894, KF923907, KF923908, KF923909, KF923910, KF923901, KF923911, KF923912, KF923913, KF923914, KF923915, KF923916, KF923917, KF923919, KF923920, KF923921, KF923922, KF923923, KY674917, KY674918, KY674920 |
| MERS | 123 | MH454272, MH822886, MH013216, KF600634, KF186567, KF600627, KF600647, KF600651, KF186565, KF186564, KU710264, KF600632, KJ156866, KT806055, KM015348, MK129253, KT225476, KT806054, KU851863, KT806051, KM027260, KF600652, KJ156881, KF600630, KT806044, KT806048, KM027255, KM027256, KM027258, KM027259, KM027261, KF958702, KF192507, KT861627, KM027257, KT374051, KT374050, KX034097, KX034099, KJ156949, KT006149, KF600628, KT806049, KT374052, KT374053, KT374054, KT374055, KU308549, KX034094, KX034095, KX034096, KX034098, KX034100, MH306207, JX869059, KU851862, KU851860, KT374056, KT374057, KT326819, NC_019843, KF745068, KT806053, KU851861, KJ156952, KJ156944, KU851864, KF600644, KF186566, KM210278, KJ156934, KM210277, KJ156874, KT806045, KC776174, KT806046, KF600613, KF600645, KC667074, KT806052, KJ156910, KT861628, KT806047, KJ156869, KM027262, KU851859, KF600612, KF600620, KP209307, KT026454, KP209313, KP209309, KP209311, KT156561, KT121577, KT121580, KT121573, KT121575, KT121572, KT121576, KP209310, KP209312, KP223131, KT121581, KT121574, KJ829365, KT121579, KT029139, KT156560, KR011266, KR011264, KR011263, KT026456, KP209308, KP209306, KT121578, KR011265, KC164505, KT026453, KT026455, KJ813439, KJ556336, KU710265 |
| Other | 128 | MN120514, MN120513, MH395139, MH432120, MK462244, MK462247, MK462248, MK462250, MK462251, MK462254, MG011341, MG011352, MG757605, MH310911, MG011343, MG011347, MG011359, MK462249, MK462253, MN723544, MK462256, MN365232, MN365233, MG011353, MH310912, MG366483, MG366882, MG912607, MG912603, MG912608, MG011360, MG011362, MK462252, MK462243, MN723542, MK462245, MK462246, MN723543, MG366883, MG912601, KX154684, MG011355, MG912595, MG912598, MH310910, MG912599, MG912604, MG011361, MG912596, MG366881, MG912602, MG912605, MG366880, MG912606, MG912597, MG912600, MK462255, KY688120, KY688123, KY688124, KY688121, KY688122, KX154690, MK039552, MK039553, MK052676, MG011351, KY673148, MG757593, MG757594, MG757595, MG757596, MG757597, MG757598, MH029552, MG757599, MG757600, MG757602, MG520075, MG520076, MG546331, MG757603, MG757604, KX154689, KX154685, KX154686, KX154687, KX154688, KX154691, KX154692, KX154693, MG011342, MG011346, MG011345, MG011349, MG011348, MG011354, MG011358, MG011357, KY581684, KY581685, KY581686, KY581687, KY581688, KY581689, KY581690, KY581691, KY581692, KY581694, MK280984, KX154694, MG757601, MG011344, MG011350, MG011356, KY581693, KY688118, MG546330, MG011340, MF000457, MF000460, MF000459, MF000458, MK483839, KY688119, MK796425, NC_038294, MH310909 |
| SARS | 42 | MK062183, MK062184, MK062181, MK062182, AY291315, MK062179, MK062180, AY274119, DQ182595, FJ882963, GU553363, GU553364, AP006557, AP006559, AP006561, AY427439, AP006560, AY310120, AY323977, AP006558, AY278489, AY390556, AY278488, AY502930, AY345986, AY345987, AY345988, AY502923, AY502929, AY278554, AY291451, AY502925, AY502928, AY502931, AY502932, AY502926, AY502927, AY502924, AY714217, AY485278, AY485277, AY282752 |
| 2019-nCoV | 34 | MT049951, LC522972, LC522973, LC522974, LC522975, MN997409, MN994467, MN994468, MT027062, MT027063, MT027064, MT044257, MN985325, MT020880, MT020881, MN975262, MT007544, MT019529, MT019530, MT019531, MT019532, MT019533, MT039873, MT066175, MT066176, MT039890, MT039888, MT039887, LC521925, MT044258, MN988713, MN938384, MN988668, MN988669 |
| Total | 590 |  |
